# *RAS* mutations in early age leukaemia modulated by *NQO1* rs1800566 (C609T) are associated with second-hand smoking exposures

**DOI:** 10.1186/1471-2407-14-133

**Published:** 2014-02-26

**Authors:** Francianne Gomes Andrade, Juliana Montibeller Furtado-Silva, Bruno Alves de Aguiar Gonçalves, Luiz Claudio Santos Thuler, Thayana Conceição Barbosa, Mariana Emerenciano, André Siqueira, Maria S Pombo-de-Oliveira

**Affiliations:** 1 Paediatric Haematology-Oncology Program, Research Centre, Instituto Nacional de Câncer - INCA, Rua André Cavalcanti, 37, Rio de Janeiro/RJ 20231-050, Brasil; 2 Great Ormond Street Hospital, London, United Kingdom; 3 Clinical Research Program, Research Centre, Instituto Nacional de Câncer, INCA, Rio de Janeiro, Brazil; 4 Universidade do Estado do Amazonas, Manaus, AM, Brazil

**Keywords:** RAS mutation, NQO1, MLL, Tobacco smoking exposures, Childhood leukaemia

## Abstract

**Background:**

Deregulation of the MAPK genes signalling caused by somatic mutations have been implied in leukaemia pathogenesis, including *RAS* mutation (*RAS*^mut^) in acute myeloid leukaemia (AML), which has been associated with intra-uterine chemical exposures. A case-case study was conducted in order to explore maternal and child exposures to tobacco smoking associations with early age leukaemia (EAL).

**Methods:**

Covariables of reference were *MLL* rearrangements (*MLL*-r), *RAS*^mut^ and *NQO1* rs1800566 (C609T). Samples from 150 acute lymphoblastic leukaemia (ALL) and 85 AML were included. Maternal exposures were assessed using a structured questionnaire with demographic, personal habits and residence history information. Restriction fragment length polymorphism and denaturing high performance liquid chromatography were used to screen *FLT3, KRAS,* and *NRAS* mutations; direct sequencing was performed to validate the results. *NQO1* polymorphism was detected by real-time allelic discrimination technique.

**Results:**

Overall, *RAS*^mut^ were detected in 28.7% of EAL cases; *BRAF*^mut^ was found only in one AML patient. Higher rate of *KRAS*^mut^ was found in ALL (30.3%) compared to AML (20.8%) with *MLL*-r; *RAS*^mut^ showed an association with second-hand tobacco smoking exposures (OR, 3.06, 95% CI, 1.03-9.07). A considerable increased risk for EAL with the combination of *RAS*^mut^ and *NQO1* 609CT (OR, 4.24, 95% CI, 1.24-14.50) was observed.

**Conclusions:**

Our data demonstrated the increased risk association between maternal smoking and EAL with *MLL*-r. Additionally, suggests that children second-hand tobacco exposures are associated with increased risk of EAL with *RAS*^mut^ modulated by *NQO1* rs1800566 (C609T).

## Background

Leukaemia that occurs in early childhood consists of one of the best models to study gene-environment exposures interactions because of the leukaemia-associated somatic mutations, the short time-frame between the environmental exposure to putative risk factors, and the clinical onset of the disease. The knowledge of leukaemogenic pathways in early age leukaemia (EAL) has improved since the consistently identified somatic gene mutations occurring during in uterus life such as *MLL* rearrangements (*MLL*-r) and *ETV6-RUNX1* fusion genes [1]. These somatic mutations might be related to the effects of error in cellular differentiation during the early life, modulated by some inherited susceptibility factors [2,3]. Environmental exposures with reproducible findings and their biological plausibility add insights into the cause of childhood leukaemia.

A variety of chemical carcinogens as cigarette smoke, hydrocarbons, medications, and methylnitrosurea [4-6] have been shown to induce mitogen-activated protein kinase (MAPK) pathway mutations in both human and animal models [6]. Deregulated MAPK signalling is commonly found in cancer cells and is often caused by mutations in *FLT3*, *RAS* family, *PTPN11*, and *BRAF*, which result in constitutive activation of pathway [7,8]. Mutations in *FLT3*, *KRAS*, *NRAS*, and *BRAF* genes have been observed with variable prevalence (5-20%) in acute myeloid (AML) and lymphoblastic leukaemias (ALL) [9-16]. Very few investigations addressed questions regarding the associations between *FLT3*, *RAS* family mutations, and parental smoking exposures in the childhood leukaemia pathogenesis [12,17,18]. The tobacco smoke content, besides nitrogen and oxygen, include toxic gases such as carbon monoxide, formaldehyde, hydrogen cyanide, acrolein, nitrogen oxides, toluene and phenolic substances that are classified as carcinogenic [19].

Additionally, some of these substances contain chemical compounds, which are substrates for the detoxification enzyme NAD(P)H: quinone oxidoreductase (*NQO1*), a flavoenzyme that detoxifies benzene metabolites, quinones, and other topoisomerase II inhibitors. The *NQO1* gene polymorphism has been associated with smoking and acute leukaemia. A study conducted in Japanese patients demonstrated that individuals with *NQO1* 609TT had a 7.6 higher risk to develop acute leukaemia [20]. In a more recent study we have observed that children with at least one *NQO1* rs1800566 (C609T) variant allele were at lower risk for developing infant AML, whereas no association was detected for ALL [21]. We have also investigated whether maternal tobacco smoking during pregnancy and lactation were associated with EAL. The association was restricted to women that reported to have smoked 20 or more cigarettes per day [22].

The aim of this study was to evaluate the association of *KRAS*, *NRAS*, *FLT3,* and *BRAF* gene mutations with EAL and also to explore whether parental smoking would be associated with acute leukaemia modulated by *NQO1* rs1800566 polymorphism.

## Methods

### Study population and samples

Diagnostic samples from 235 Brazilian children with acute leukaemia (ALL or AML) aged ≤24 months were selected for molecular analyses depending upon availability of biological material. Cases were included when the diagnostic material had good-quality DNA isolated from bone marrow aspirates with at least 50% of blast cells [23-25].

Cases were diagnosed according to morphology, immunophenotyping, and molecular characterization. The exclusion criteria were patients with clinical phenotypes resulting from genetic syndromes (Down, Ataxia Teleangectasia, Boom, Noonan, chromosome 7 monossomy) and/or acute leukaemia with biological material ≤49% of blast cells. The absence of a confirmed diagnosis and inaccessibility to the biological mother were also exclusion criteria for study enrolment.

All co-participants approved the study according to Brazilian National Research and Ethics Committees (CONEP) followed by the Instituto Nacional de Câncer Research and Ethics Committees (CEP) under the registry: (CEP #005/06, Multi-institutional Study of Infant Leukemia: Contribution of Immunomolecular Markers in Distinguishing Different Etiopathogenic Factors); and CEP #024/10-CONEP #707/2010: Genomic study of infant leukemia with MLL rearrangements). Written consent was obtained from the parents for both polymorphisms and maternal questionnaire procedures.

### RNA and DNA extraction

RNA was purified using TRIZOL reagent (Invitrogen, CA, USA), according to the manufacturer’s instructions. cDNA was synthesized applying the transcriptase reverse enzyme. The cDNAs quality was verified throughout *GAPDH* gene amplification. The DNA extraction was performed using the QIAmp DNA Blood Mini Kit (Qiagen, CA, USA) according to the manufacturer’s instructions.

### Reverse transcriptase polymerase chain reaction (RT-PCR) for identifying fusion genes

All patients diagnosed with one of the childhood ALL or AML subtypes were screened for common genetic abnormalities (*ETV6-RUNX1, TCF3-PBX1, BCR-ABL1, AML1-ETO, CBFβ-MYH11, PML-RARa*) according to the BIOMED-1 Concerted Action [26].

### *MLL* rearrangements

RT-PCR was performed to identify the more common fusion transcripts of *MLL* gene (*AFF1/AF4, MLLT3/AF9, MLLT1/ENL, MLLT10/AF10*, and *ELL*), according to methods previously described [27]. For patients younger than 12 months, an extended detection of *MLL*-r was performed with fluorescence *in situ* hybridisation (FISH) with a commercial DNA probe (LSI MLL Dual Colour Break Apart Rearrangement Probe, Vysis Inc. IL, USA) according to manufacturer’s instructions.

### *RAS* gene amplifications and detection of mutations

Codons 12 and 13 of *KRAS* and *NRAS* genes were amplified of genomic DNA according to methods described by Bornholdt *et al.* and Liang *et al.,* respectively [10,28]. Both PCR reactions were carried out on a GeneAmp® PCR System 9700 Thermal Controller (Applied Biosystems, Foster City, CA). Two different techniques were used to detect point mutations of *K*- and *N*-*RAS:*

1- For *KRAS* amplification, the cycling conditions followed the conditions of denaturation at 94°C for 1 min, taken through 35 cycles at 94°C for 30 sec, 60°C for 45 sec, 72°C for 45 sec; and a final incubation at 72°C for 10 min. All PCR products were visualized using a 1.5% agarose gel and ethidium bromide staining. The restriction fragment length polymorphism (RFLP) was performed to detect *KRAS* mutations. For codon 12, 5–16 μl of PCR product were treated with the restriction enzyme *BstNI* (Biolabs, New England, UK); PCR products of codon 13 were treated with *PflMI*. The digested PCR fragments were visualized on a 3% agarose gel. Two types of negative controls were used: blank reaction without DNA addition and amplification reaction with DNA from a healthy person. For positive controls, in the codon 12 assay, sample from a juvenile myelomonocytic leukaemia which contains a homozygous GGT → AGT mutation was used; for codon 13, the MDA-MB231 cell line with a heterozygous GGC → GAT mutation was used.

2- Denaturing high-pressure liquid chromatography (dHPLC) was used to screen *NRAS* mutations by using a Transgenomic Wave machine (Transgenomic, NE, UK). The PCR assay was performed using 70-100 ng of genomic DNA with 0,625U of Taq DNA polimerase (Invitrogen, Carlsbad, CA), 1x PCR buffer (50 mM KCl, 20nM Tris–HCl pH 8,4); 1,5 mM MgCl_2_; dNTPs 0,2 mM and 0.4 μM each forward and reverse primer. *PCR* amplification with the protocol of initial preheating at 94°C for 5 min, 40 cycles of denaturation at 94°C for 1 min, annealing at 57°C for 1 min, and 72°C for 1 min; and a final extension at 72°C for 10 min. The fragments were visualized on a 1.5% agarose gel staining with ethidium bromide. Prior to dHPLC analysis these products were denaturated at 95°C for 5 min and subsequently cooled down using a temperature’s gradient until 50°C using a thermal cycler. Forty μL of the PCR product were injected into the DNASep HT column for analysis. The products were eluted at a flow rate constant of 1,5 mL/min with a linear gradient of acetonitrile and annealing profiles for PCR products were determined by the Navigator software (Transgenomic, NE, UK) based on the size and GC content of the amplicons.

Finally, mutations found in *K*- and *N*-*RAS* throughout both methods (RFLP and DHPLC) were confirmed by DNA sequencing according to manufacturer’s instructions (BigDye® Terminator v3.1 Cycle Sequencing Kit, Applied Biosystems, CA, USA) in the ABI3130xl Genetic Analyzer (Applied Biosystems, CA, USA) as previously described [28].

### Mutation detection of *BRAF* gene

Detection of mutations of *BRAF* gene was performed using pyrosequencing (PSQ), which is based on ‘sequencing by synthesis’ principle [29]. PCR reaction using the PyroMark Q24 BRAF Kit (Qiagen, CA, USA) allowed the amplification of exons 11 and 15 of the gene. The biotinylated PCR products was immobilized onto streptavidin-coated sepharose beads (Amersham Biosciences, NJ, USA) and processed to obtain a single strand DNA through the PyroMark Q24 Vacuum Prep Workstation (Qiagen, CA, USA). The strands were separated using 0.1 mol/l NaOH. The supernatant was then discarded and 0.3 μΜ of the PSQ primer added annealed to the captured strand and incubated at 80°C for 2 minutes on the PSQ plate. The biotinylated DNA with annealed sequence primer was released from the streptavidin surface according to the manufacturer’s instructions (Qiagen, CA, USA). The primed single-stranded DNA templates were subjected to real-time sequencing of the region surrounding codons 464–469 in exon 11 and codon 600 in exon 15. The optimal nucleotide dispensation order was determined by assessing the theoretical outcome of a number of dispensation orders. Identified mutations were confirmed by PSQ of an independent PCR [30].

### Screening of *FLT3* alterations

The presence of *FLT3* mutations were screened according to methods previously described [31]*. FLT3* D835 point mutations were detected by genomic amplification of *FLT3* exon 17 and further digestion with *EcoRV* enzyme. PCR products were separated by electrophoresis in agarose gel stained with ethidium bromide. *FLT3* internal tandem duplication (ITD) was screened by amplification of the juxtamembrane domain in exons 11 and 12 and further visualized in 3% agarose gel. Samples with altered pattern and available biological material were also sequenced.

### *NQO1* Genotyping

The EAL potential risk association with *NQO*1C609T genotypes were previously investigated in a case–control study [21], in which, a control group of selected children without malignancies, from the same regions as the cases were age-matched. *NQO1* rs1800566 (C609T) polymorphism was detected by allelic discrimination using TaqMAN® probes (TaqMAN® SNP Genotyping Assays; Applied Biosystems, Foster City, CA, USA) using the BioRad C1000 Thermal Cycler (CFX96 Real Time System). The primer/probe system and the reaction protocol used are described elsewhere [21].

### Collection of information and assessment of smoking exposure

Cases were assessed throughout the study [Multi-institutional Study of Infant Leukemia: Contribution of Immunomolecular Markers in Distinguishing Different Etiopathogenic Factors] that focuses on the investigation of EAL [22,23]. Participants were ascertained from different Brazilian regions (shown in Additional file 1: Table S1) from January 2000 to December 2010. Exposure information was obtained with the aid of a well-structured questionnaire throughout face-to-face interview with case and control mothers, after signing a written informed consent. Briefly, the mother’s questionnaires included questions about family income, maternal age, education level, illness previous history, medications use, occupation, personal recreate habits, and the child’s birth characteristics. The exposure assessment regarding smoking was first determined by the qualitative analysis (yes/no) during the three months before the index pregnancy, the three trimesters of the pregnancy, as well as, after birth during the breastfeeding period. Regarding the affirmativeness of maternal smoking antecedents and/or other person living at home, additional information were included, such as, the usual amount of daily smoked cigarettes during preconception, pregnancy and breastfeeding. Usual smoking frequency at these time windows were also collected as: no primary hand smokers; moderate smokers as less than 20 smoked cigarettes per day; and heavy smokers as 20 or more smoked cigarettes per day [22].

### Statistical analyses

The design is a case-case study in which ALL were compared with AML according to distribution of gene mutations, age at onset of the disease, parental’s demographics and maternal exposures to tobacco smoking. The Pearson’s chi-square and Fisher exact tests and child profile were used to assess differences in discrete variables. Crude and adjusted odds ratio (OR) and their 95% confidence intervals (CI) for selected variables (age, gene mutations, leukaemia subtype) were assessed using unconditional logistic regression in order to estimate the magnitude of associations. Association tests between biological variables and parental smoking habits before, during and after pregnancy were performed**.** The *NQO1* status was considered as at least one T allele variant for analysis [21].

Following previous studies exploring the association of different variables with the occurrence of paediatric leukaemia [17], a multivariable log-linear model was built including the variables *RAS* mutation, *NOQ1609CT*, presence of *MLL*-r and presence of smoker within the household (SMOKER) and two-by-two interaction terms between them. Full log-linear model*:* Log(count) = a + b1(Var1) + … + b4(Var4) + c1(Var1 * Var2) + c2(Var1 * Var3) + c3(Var1 * Var4) + d1(Var2 * Var3) + d2(Var2 * Var4) + e1(Var3 * Var4). A sub model without each of the interaction terms was compared to the full model containing all the pairwise interactions by performing the log-likelihood test and generating a P- value, allowing testing if the pairwise associations between the variables was relevant in the study population. The log-linear analysis is an extension of the Pearson’s χ^2^ test, consisting of and additive part of each variable main effects combined with the pairwise multiplicative interaction terms. For all analyses, the *p* values <0.05 were considered statistically significant.

An estimate of overall survival (OS) was determined using the Kaplan–Meier and log rank tests in order to verify the association of *RAS*^mut^ and *RAS* wild-type (*RAS*^wt^) in the children outcome. Patients lost to follow-up were censored at their date of last known contact. The statistical Software SPSS Version 18.0 was used (SPSS Inc, Chicago, IL, USA).

## Results

### Clinical characteristics of the study population

The demography and clinical characteristics of EAL are shown in Table 1. There were 150 ALL and 85 AML patients. The majority of them were infants (≤ 12 months) and the median age at diagnosis was 11 months; with slight predominance of male, although without statistical significance. The difference of proportion of children with very high white blood cell (WBC) count was statistically significant (p = 0.014); with ALL cases presenting very high WBC count compared to AML. Regarding immunophenotype, ALL cases were mainly pro-B (CD10-) subtype, whereas the myelomonocytic differentiated leukaemia subtype (FAB M4-M5) was present in 63.2% of the AML (data not shown).

**Table 1:** Demography and clinical characteristics of early age acute leukaemia subtypes, Brazil, 2000-2010

	**Total, n (%)**	**ALL (%) (n = 150)**	**AML (%) (n = 85)**	***p***
**Age (months)**				
≤12	120 (51.1)	82 (54.7)	38 (44.7)	0.14
13-24	115 (48.9)	68 (45.3)	47 (55.3)	
**Gender**				
Male	131 (55.7)	78 (52.0)	53 (62.4)	0.12
Female	104 (44.3)	72 (48.0)	32 (37.6)	
**Skin colour**				
White	139 (59.9)	92 (61.7)	47 (56.6)	0.44
Non-White	93 (40.1)	57 (38.3)	36 (43.4)	
**WBC (x10**^**9**^**/L)**				
≤50	106 (46.5)	59 (40.4)	47 (57.3)	0.01
>50	122 (53.5)	87 (59.6)	35 (42.7)	
**Chromosomal alterations**				
*MLL*^*r*^	102 (77.3)	73 (78.5)	29 (74.4)	0.006
*ETV6-RUNX1*	7 (5.3)	7 (7.5)	0 (0.0)	
Hyperdiploidy	6 (4.5)	6 (6.5)	0 (0.0)	
Others	17 (12.9)	7 (7.5)^a^	10 (25.6)^b^	
***RAS***^**c**^				
Wild-type	175 (74.5)	105 (70.0)	70 (82.4)	0.03
Mutated	60 (25.5)	45 (30.0)	15 (17.6)	
***BRAF***				
Wild-type	124 (99.2)	61 (100.0)	63 (98.4)	1.00
Mutated	1 (0.8)	0 (0.0)	1 (1.6)	
***FLT3***				
Wild-type	148 (93.1)	88 (91.7)	60 (95.2)	0.52
Mutated	11 (6.9)	8 (8.3)	3 (4.8)	
***NQO1 *** **(rs1800566)**^**d**^				
CC	97 (54.2)	67 (53.6)	30 (55.6)	0.91
CT	70 (39.1)	50 (40.0)	20 (37.0)	
TT	12 (6.7)	8 (6.4)	4 (7.4)	

^a^Four pre-B ALL cases presenting the fusion transcript *TCF3-PBX1*, one pre-B ALL case presenting the cytogenetics t(2;14)(p10;q23), one pre-B ALL case presenting the cytogenetics t(5;15)(q12;q13) and one common ALL case presenting complex karyotype. ^b^One M4-AML presenting *RUNX1-RUNX1T1* fusion transcript. Five M4-AML cases presenting *CBFβ-MYH11* fusion transcript. One M3-AML presenting *PML-RARa* fusion transcript. One M0-AML, one M7-AML case and one AML case not otherwise specified presenting complex karyotype. AML subtypes according to FAB classification; ^c^Mutations in either *KRAS* or *NRAS*. ^d^Genotype frequencies of *NQO1* polymorphism. ALL: acute lymphoblastic leukaemia; AML: acute myeloid leukaemia; n: number of cases; WBC: white blood cell; *MLL*^*r*^: rearranged *MLL*.

In 102 out of 138 cases (73.9%) the presence of an *MLL*-r could be detected; the *MLL* status indeterminate category includes those with insufficient biological material (n, 24); the remaining 109 cases had the *MLL* status defined as negative. Somatic chromosomal aberrations, such as hyperdiploidy (n, 10), translocation with the fusion transcript *ETV6-RUNX1* (n, 7) *TCF3-PBX1* (n, 4), t(2;14)(p10;q23) and t(5;15)(q12;q13) and complex karyotype (n, 3) were found in ALL cases older than 12 months of age at the diagnosis. *RUNX1-RUNX1T1* and *CBFβ-MYH11* fusion transcripts were found in 6 AML cases; one child with AML-M3 presented *PML-RARa* fusion transcript.

### MAPK gene mutations in EAL

*RAS* mutations were found in 60 (25.5%) of the acute leukaemia cases, whereas *FLT3* mutations were found in 11 patients (6.9%) predominantly among ALL and not statistically significant. One hundred twenty-five cases were successfully analysed for *BRAF* mutations and only one AML case harboured a mutation located at c.1799 T > A (V600E). Concurrent mutations in *FLT3* and *RAS* in the same patient were detected in six patients; two of these cases harboured an *MLL*-*AFF1* fusion gene. The demographic characteristics and the distributions of *KRAS, NRAS,* and *FLT3* mutations were not statistically different according to age strata (shown in Additional file 2: Table S2). *KRAS* mutations (*KRAS*^mut^, n = 47) represented 20.0% and *NRAS* (n, 14) 16.1%. While *FLT3* mutations (n, 11) were equally distributed in infants and children older than 12 months, *KRAS*^mut^ was strongly associated with age less than 12 months and *MLL-r* (p = 0.001), as shown in Additional file 3: Table S3.

We have tested acute leukaemia subtypes associated with *MLL-r* and the frequencies of *RAS* and *FLT3* mutations in EAL (Additional file 4: Table S4). *KRAS*^mut^ were detected in 28.6% of patients with infant B-precursor ALL harbouring *MLL*-r and in 20.7% of patients with AML and *MLL*-r. The association of *KRAS*^mut^ and *MLL*-r was OR, 2.16, 95% CI, 1.07-4.38; children with AML were more prone to have *MLL*-r with *KRAS*^mut^, although not statistically significant. The frequencies in *FLT3* mutations (n, 3) in ALL and in AML (n, 2) were not significant to be included in further analysis. Only one case presented a *BRAF* mutation, demonstrating that is a rare alteration in EAL.

The OS analysis showed no differences between the *RAS*^mut^ and *RAS*^wt^ among ALL and AML according to patient age at diagnosis (Additional file 5: Figure S1). *RAS*^mut^ cases presented a poorer median survival outcome (14.0 months, 95% CI, 4.01-23.93 for patients aged ≤12 months and 24.6 months, 95% CI, 0–72.46 for patients aged 13–24 months) in ALL (Additional file 5: Figure S1A and C) similar to *MLL*-r as a single genetic aberration in ALL cases (12.2 months, 95% CI, 2.63-21.77 and 41.0 months, 95% CI, 2.34-79.73), shown in Additional file 5: Figure S1B and D. Among AML cases, the combination of *RAS*^mut^ and *MLL*-r showed a similar poorer outcome; high OS for *RAS*^mut^ cases presenting *MLL* wild type (*MLL*^wt^) was also found (Additional file 5: Figure S1E and F).

### *NQO1* gene polymorphism

The distributions of the *NQO1* rs1800566 (C609T) allele frequencies in early age ALL and AML are shown in Table 1 and the frequency association between *RAS* mutations and *NQ01* rs1800566 (C609T) genotype are presented in Additional file 6: Table S5. There was an association with *NQO1*T609T and *RAS* mutation (OR, 1.60 95% CI 0.47-5.44) without statically significance. The association of *NQO1* 609CT, *RAS* and *MLL* status in these settings of childhood leukaemia were further tested and results are presented in Additional file 7: Table S6. The effect of at least one *NQO1* rs1800566 (C609T) variant presented an increased risk association with ALL with *MLL*-r, although not statistically significant (p = 0.09).

### Maternal exposures to tobacco smoking and *RAS* mutation

Information on the lifelong length of tobacco exposure, smoking during pregnancy and second-hand smoking were obtained and reported previously; no dose–response effect was found [22]. The cohort with the addition of molecular markers was re-visited. Analysis of demographic and clinical distribution of cases according to available data from mother interview showed no statistical differences between variables (Additional file 1: Table S1);

The risk association of tobacco smoking exposure during pregnancy, with the *RAS* status, *NQO1* polymorphism, and EAL with *MLL-r* are shown in Table 2. Maternal tobacco smoking ever smoked, maternal smoked 3 months before and, maternal smoked during the index pregnancy were increased associated with *MLL-r* (OR, 2.90, 95% CI, 1.09-7.71).

**Table 2:** **The risk of association of smoking exposures during pregnancy with *RAS *** **status, *NQO1 *** **and *MLL *** **rearrangements, EAL cases, in a case-case analysis Brazil, 2000-2010**

	***RAS***^***wt***^** n (%)**	***RAS***^***mut***^** n (%)**	**OR (95% CI)**	***NQO1***^***wt ***^**n (%)**	***NOQ1609CT***^**a **^**n (%)**	**OR (95% CI)**	***MLL***^***wt***^** n (%)**	***MLL***^***r***^** n (%)**	**OR (95% CI)**
**Mother ever smoked**									
No	55 (65.5)	24 (60.0)	1^b^	39 (57.4)	40 (71.4)	1^b^	45 (76.3)	30 (52.6)	1^b^
Yes	29 (34.5)	16 (40.0)	1.26 (0.58-2.75)	29 (42.6)	16 (28.6)	0.54 (0.25-1.14)	14 (23.7)	27 (47.4)	2.89 (1.31-6.40)
**Mother smoked 3 months before pregnancy**									
No	59 (70.2)	28 (70.0)	1^b^	44 (64.7)	43 (76.8)	1^b^	48 (81.4)	34 (59.6)	1^b^
Yes	25 (29.8)	12 (30.0)	1.01 (0.44-2.30)	24 (35.3)	13 (23.2)	0.55 (0.25-1.23)	11 (18.6)	23 (40.4)	2.95 (1.27-6.85)
**Mother smoked during pregnancy**									
No	67 (79.8)	31 (77.5)	1^b^	52 (76.5)	46 (82.1)	1^b^	52 (88.1)	41 (71.9)	1^b^
Yes	17 (20.2)	9 (22.5)	1.14 (0.46-2.85)	16 (23.5)	10 (17.9)	0.71 (0.29-1.71)	7 (11.9)	16 (28.1)	2.90 (1.09-7.71)
**Someone in the house ever smoked**^**c**^									
No	46 (59.0)	16 (42.1)	1^b^	38 (62.3)	24 (43.6)	1^b^	30 (53.6)	29 (53.7)	1^b^
Yes	32 (41.0)	22 (57.9)	1.98 (0.90-4.34)	23 (37.7)	31 (56.4)	2.13 (1.02-4.49)	23 (46.4)	25 (46.3)	1.00 (0.47-2.11)

^a^*NQ01* genotype status included allele variants CT and TT. ^b^1 as a reference. ^c^Mothers reported the presence of someone smoking at home during the index pregnancy (second-hand smoking). *MLL*^*wt*^: Wild type *MLL*; *MLL*^***r***^: Rearranged *MLL*; n: number of cases; wt: wild type.

Moreover, 54 (45.5%) mothers reported the presence of someone smoking at home during the index pregnancy (second-hand smoking). No risk associations with mothers who ever smoked (before and/or during pregnancy) were observed with *RAS* status and *NQO1* polymorphism. However, an increased risk association was observed with *NQO1609CT* and the presence of someone smoking at home during the EAL pregnancy (OR, 2.13, 95% CI, 1.02-4.49).

The risk association of smoking exposures during pregnancy combining *NQO1* and *RAS* status was tested (Table 3). The presence of someone in the house who ever smoked (second-hand smoking) in children with EAL with *NQO1* wild-type*/RAS*^*mut*^ (OR, 3.06, 95% CI, 1.03-9.07); the association of second-hand smoking during pregnancy and children with *NQO1* rs1800566 (C609T) polymorphism increased 2.97 folds the risk of developing leukaemia (95% CI, 1.16-7.60); *RAS*^mut^ and the presence of at least one variant allele of *NQO1* rs1800566 (C609T) showed an increased risk developing leukaemia (OR, 4.24, 95% CI, 1.24-14.50). No associations were observed with *FLT3* mutations and maternal exposures (data not shown). The results of the multivariable log-linear model and to measure the association with selective variables shown in Table 4, confirmed the association of *RAS*^mut^ and second-hand smoking and the *NQO1* rs1800566 (C609T) polymorphism and second-hand smoking as EAL risk factors.

**Table 3:** **The risk of association of smoking exposures during pregnancy, combining *NQO1 *** **and *RAS *** **status, in a case-case analysis Brazil, 2000-2010**

	***NOQ1***^***wt***^ ***/RAS***^***wt ***^**n (%)**	***NOQ1***^***wt***^ ***/RAS***^***mut ***^**n (%)**	**OR (95% CI)**	***NOQ1609CT***^**a**^ ***/RAS***^***wt ***^**n (%)**	**OR (95% CI)**	***NOQ1609CT***^**a**^ ***/RAS***^***mut ***^**n (%)**	**OR (95% CI)**
**Mother ever smoked**							
No (n,79)	26 (57.8)	13 (56.5)	1^b^	29 (74.4)	1^b^	11 (64.7)	1^b^
Yes (n,45)	19 (42.2)	10 (43.5)	1.05 (0.38-2.90)	10 (25.6)	0.47 (0.19-1.20)	6 (35.3)	0.75 (0.24-2.38)
**Mother smoked 3 months before pregnancy**							
No (n,87)	29 (64.4)	15 (65.2)	1^b^	30 (76.9)	1^b^	13 (76.5)	1^b^
Yes (n,37)	16 (35.6)	8 (34.8)	0.97 (0.34-2.77)	9 (23.1)	0.54 (0.21-1.42)	4 (23.5)	0.56 (0.16-2.00)
**Mother smoked during pregnancy**							
No (n,98)	35 (77.8)	17 (73.9)	1^b^	32 (82.1)	1^b^	14 (82.4)	1^b^
Yes (n,26)	10 (22.2)	6 (26.1)	1.24 (0.39-3.97)	7 (17.9)	0.77 (0.26-2.25)	3 (17.6)	0.75 (0.18-3.14)
**Someone in the house ever smoked**^**c**^							
No (n,62)	28 (71.8)	10 (45.5)	1^b^	18 (46.2)	1^b^	6 (37.5)	1^b^
Yes (n,54)	11 (28.2)	12 (54.5)	3.06 (1.03-9.07)	21 (53.8)	2.97 (1.16-7.60)	10 (62.5)	4.24 (1.24-14.50)

^a^*NQ01* genotype status included allele variants CT and TT. ^b^1 as a reference. ^c^Mothers reported the presence of someone smoking at home during the index pregnancy (second-hand smoking). N: number of cases; wt: wild-type; mut: mutation.

**Table 4:** Multivariable log-linear model and selective associations in leukaemia cases

**Measure of association terms**	**DF**	**χ**^**2**^**-value**^**a**^	**P-value**^**b**^
RAS*NQO1^c^	1	1.11	0.29
RAS*MLL	1	0.15	0.70
RAS*SMOKER	1	4.61	0.03
NQO1*MLL	1	0.24	0.62
NQO1*SMOKER	1	4.84	0.03
MLL*SMOKER	1	0.12	0.73

^a^χ^2^-value from the log-likelihood ratio test comparing the submodel without the interaction term with the full model. ^b^P-value from the log-likelihood ratio test – Low values indicate that the interaction between the two variables are meaningful in the given population. ^c^*NQO1* genotype status included allele variants CT and TT. SMOKER: Presence of any smoker in the household; DF: Degrees of Freedom.

## Discussion

Childhood leukaemia is resultant of a multiple causation factors. The exogenous or endogenous exposures, the intrinsic susceptibility of the child given by single-nucleotide polymorphism may increase the chances of childhood leukaemia [3,8]. This unique study group comprises a series of EAL children with ALL and AML in which somatic mutations arise at the stem-cell level during fetal hematopoiesis [2,31]. EAL represents an epidemiological particular group, because transplacental exposures to DNA damaging substances is proposed to be associated with an increased risk of such leukaemias [23,32-34]. Very few studies have tested the association between smoking and the presence of selected genetic characteristics in childhood.

First of all, the analyses took into account the relatively high prevalence of MAPK mutations (mainly *RAS*^mut^ 25.5%) in these EAL settings associated with *MLL-r*. The prognostic value *RAS*^mut^ in childhood leukaemia with or without *MLL*-r was described and demonstrated the poor risk associations [10,32]. This effect was also demonstrated in the present setting, despite of week statistical power.

Albeit, the frequency of *KRAS*^mut^ is higher in patients aged ≤12 months with *MLL*-r than in patients with *MLL*^wt^, few studies explored the association of *RAS*^mut^, as a prenatal event in addition to *MLL*-r leukaemogenesis [35].

Previous studies suggest that *RAS*^mut^ may be associated with chemical exposures. A strong statistical association between *RAS*^mut^ and AML patients with prior exposures to chemotherapy and X-ray was described [9]. Mutagenic chemicals from maternal smoking cross the placenta enhancing the plausibility of an effect of parental tobacco smoking on childhood leukaemia risk [36]. Our data showed a strong association between *RAS*^mut^ and the presence of someone smoking in the house during the pregnancy or the early life of the child with acute leukaemia.

In a previous study, maternal tobacco smoking during pregnancy was reported by 17.5% of case mothers and 20.6% of controls and no association with ALL or AML was observed [22]. This null association between maternal smoking and EAL risk regardless the period of exposure during pregnancy is consistent with the literature [37]. All analysed data were dependent on maternal report, which may have introduced incorrect exposure estimates, with some exposures possibly being under-reported [38]. It is important to remark that an increased risk association was observed with heavy smoker mothers. In these former studies, the presence of somatic *RAS*^mut^ and/or genetic susceptibility was not attempted to.

Someone in the house ever smoked, but mothers, is characterized by relatives such as husband, grandparents and/or nanny “smokers” living in the house and taking care of the child. The interpretation for this counter intuitiveness [first” hand smoke has no effect whereas second hand smoke does] would be considered (or implied) that some mothers might had denied being a smokers due to guilt. This is one pitfall of epidemiological studies based on questionnaire responses. Censured topics and variables such drug and/or tobacco users values are depending upon the commitment of different actors under social pressures. However, the biologic plausibility for the present findings is that cigar metabolites compounds carcinogens’ substances such phenols, formaldehyde, toluene and others co-carcinogens (catechol and related compounds), toxic agents (acrolein and other aldehydes) and free radical species (nitric oxide and others) that are spread in the air by the smokers are contaminants with DNA damage power. The carcinogenesis pathways of these substances have already been determined [19]. Metayer et al. have just published that children with history of paternal smoking combined with postnatal passive smoking were at 1.5 fold-increased risk of ALL [39]. Other factors such as genetic susceptibility that modulate the risk need to be considered.

Molecular epidemiology suggests that the risk conferred by gene variants have decreased the expression of specific products, but contribute to the intrinsic vulnerability of immature cells to initiate an abnormal clone. In this context, the polymorphism of *NQO1* gene involved in benzene activation and participating in benzoquinone detoxification and reactivating benzene intermediates, might contribute to outgrowth extrinsic effect in exposed cells [20]. *NQO1* rs1800566 heterozygous individuals (C/T) have intermediate enzyme activity, and homozygotes for the variant allele (T/T) are deficient in NQO1 activity [40]. According to some studies (mainly of subjects of European white ancestry), lower NQO1 activity was associated with an increased risk of infant ALL carrying *MLL/AFF1* fusion genes [41-44]. The differences in the distribution of *NQO1* rs1800566 (C609T) according to age strata in EAL and control groups in Brazilian settings were tested and the genetic susceptibility data regarding *NQO1* variants are described elsewhere [21]. In brief, an increased crude risk for *NQO1* C609T was detected in children older than 1 year, although this association lacked statistical significance (OR, 1.83, 95% CI, 0.82-4.06), whereas, a protective effect of the *NQO1* C609T variant was found for infants with *MLL* germ line acute leukaemia (OR, 0.36, 95% CI, 0.16-0.81) [21].

The observation that the combination of *RAS*^mut^ and *NQO1* variants showed a crescent association with the presence of someone smoking in the house suggests the synergistic effect of both genetic alterations contributing for leukaemogenesis. At such age strata, with a short postnatal life span of exposures, the importance of exploring gene mutations and environmental exposures is of paramount importance, because it allows to speculate that despite of the timing of tobacco smoked during pregnancy the dose-effect was modulated by genetic susceptibility with the potential of causing EAL. These results add evidence to our hypothesis that *RAS*^mut^ might be consequence of hazardous exposures in individual with genetic susceptibility.

The current investigation has some limitations, as consequence of case-case study in such rare disease. First, the reduced numbers of children with low frequency of maternal exposures make odds ratios unstable and may have incurred in insufficient statistical power to detect significant differences. Second, the selection of cases due to biological material available may have introduced selection bias. However, the comparison of the analysis of the biological samples from children with and without maternal interview was similar. Another weakness of our study that should be pointed out is the missing assess of parental occupational exposures that involve chemical substances such as dyes, polycyclic aromatic hydrocarbons, organic powder/industrial dust, in which, *KRAS*^mut^ was observed to be strongly associated.

On the other hand, the study presents strong points. At first, it allowed the collection of maternal data exposure to smoking in a very rare disease setting such as infant leukaemia and combines the results with somatic gene mutations and inherited predisposition via gene variant related to smoking exposure.

Finally, to our knowledge, this is the first study exploring the association between maternal exposure to tobacco smoking during pregnancy and *RAS* and *NQO1* polymorphism in EAL with *MLL*-r.

## Conclusions

The present data demonstrated the increased risk association between maternal smoking and EAL with *MLL*-r. Additionally, suggests that second-hand tobacco smoking exposures are associated with increased risk of EAL with *RAS*^mut^ modulated by *NQO1* rs1800566 (C609T). The significant associations found here could guide the design of other etiological studies in childhood leukaemia, emphasizing the critical role of genetic susceptibility with somatic mutations in the mechanistic pathway leading to leukaemia in childhood.

## Abbreviations

ALL: Acute lymphoblastic leukaemia; AML: Acute myeloid leukaemia; CI: Confidence intervals; dHPLC: Denaturing high-pressure liquid chromatography; EAL: Early age leukaemia; FISH: Fluorescence in situ hybridisation; ITD: Internal tandem duplication; MAPK: Mitogen-activated protein kinase; *MLL-r*: *MLL* rearrangements; MLL^wt^: MLL wild type; NQO1: Detoxification enzyme NAD(P)H: quinone oxidoreductase; OR: Odds ratio; OS: Overall survival; PSQ: Pyrosequencing; RAS^mut^: RAS mutation; RAS^wt^: RAS wild-type; RFLP: Restriction fragment length polymorphism; RT-PCR: Reverse transcriptase polymerase chain reaction; WBC: White blood cell.

## Competing interests

The authors declare that they have no competing interests.

## Authors’ contributions

FGA, JMFS, BAAG and ME analyzed and interpreted the data and wrote the manuscript. FGA, JMFS, BAAG and TCB performed the laboratory work of this study. FGA, JMFS and LCST participated in the statistical analysis. MSPO contributed to the conception and the design of the study, revised the article critically, and made the final approval of the version to be submitted. BCSGIAL contributed with samples. All authors’ read and approved the final manuscript.

## Pre-publication history

The pre-publication history for this paper can be accessed here:

http://www.biomedcentral.com/1471-2407/14/133/prepub

## Supplementary Material

Additional file 1: Table S1 Demographic and clinical distribution of variables analysed in childhood acute leukaemia according to maternal enquires, Brazil, 2000-2010. Click here for file

Additional file 2: Table S2 Demography and laboratorial characteristics of earl age leukaemia according to age groups, Brazil. Click here for file

Additional file 3: Table S3 The demography and laboratorial differences in the distribution of *RAS* mutations, according to age strata, Brazil, 2000-2010. Click here for file

Additional file 4: Table S4 The association of *RAS* and *FLT3* mutations in early age leukaemia according to *MLL* status, Brazil 2000-2010. Click here for file

Additional file 5: Figure S1 Overall survival of *RAS* mutations (*K-* or *N-RAS*) and *RAS* (*K-* or *N-RAS*) wild-type among ALL and AML according to patients age at the diagnosis. (A) *RAS* mutations in ALL cases aged ≤12 months. (B) *RAS* mutations and *MLL* rearrangements in ALL cases aged ≤12 months. (C) *RAS* mutations in ALL cases aged 13–24 months. (D) *RAS* mutations and *MLL* rearrangements in ALL cases aged 13–24 months. (E) *RAS* mutations in AML cases aged ≤24 months. (F) *RAS* mutations and *MLL* rearrangements in AML cases aged ≤24 months. ALL: acute lymphoblastic leukaemia; AML: acute myeloid leukaemia. Click here for file

Additional file 6: Table 5S The frequency association between *RAS* mutations and *NQ01* genotype in EAL. Click here for file

Additional file 7: Table S6 The *NQO1*, *RAS* and *MLL* status in childhood leukaemia, Brazil 2000-2010. Click here for file

## Fundings

MSPO is supported by CNPq research scholarship #302423/2010-9 and the project was granted FAPERJ E-26/101.562/2010.
