# Supplementary material for: RAS mutations in early age leukaemia modulated by NQO1 rs1800566 (C609T) are associated with second-hand smoking exposures
Source: BMC Cancer. 2014 Feb 26;14:133. doi: 10.1186/1471-2407-14-133 (PMC3946262; doi:10.1186/1471-2407-14-133)
Supplement: Additional file 1: Table S1 — Demographic and clinical distribution of variables analysed in childhood acute leukaemia according to maternal enquires, Brazil, 2000-2010. [file 1471-2407-14-133-S1.doc]

**Additional File 1: Table S1. Demographic and clinical distribution of variables analysed in childhood acute leukaemia according to maternal enquires, Brazil, 2000-2010**

|  |  | **Maternal Interview** | |  |
| --- | --- | --- | --- | --- |
|  | **Total, n(%)** | **Yes, n(%)** | **No, n(%)** | ***p*** |
| **Age (months)** |  |  |  |  |
| ≤12 | 120 (51.1) | 77 (52.7) | 43 (48.3) | 0.51 |
| 13-24 | 115 (48.9) | 69 (47.3) | 46 (51.7) |  |
| **Gender** |  |  |  |  |
| Male | 131 (55.7) | 84 (57.5) | 47 (52.8) | 0.47 |
| Female | 104 (44.3) | 62 (42.5) | 42 (47.2) |  |
| **Skin colour** |  |  |  |  |
| White | 139 (59.9) | 90 (62.5) | 49 (55.7) | 0.30 |
| Non-White | 93 (40.1) | 54 (37.5) | 39 (44.3) |  |
| **Birth weight (g)** |  |  |  |  |
| <2.500 | 11 (9.2) | 11 (9.7) | 0 (0.0) | 0.19 |
| 2.501-3.499 | 61 (50.8) | 59 (52.2) | 2 (28.6) |  |
| ≥3.500 | 48 (40.0) | 43 (38.1) | 5 (71.4) |  |
| **WBC (x10^9^/L)** |  |  |  |  |
| ≤50 | 106 (46.5) | 69 (48.9) | 37 (42.5) | 0.34 |
| >50 | 122 (53.5) | 72 (51.1) | 50 (57.5) |  |
| ***MLL* status** |  |  |  |  |
| Rearranged | 102 (48.3) | 64 (48.1) | 38 (48.7) | 0.93 |
| Wild-type | 109 (51.7) | 69 (51.9) | 40 (51.3) |  |
| ***KRAS*** |  |  |  |  |
| Wild-type | 188 (80.0) | 114 (78.1) | 74 (83.1) | 0.34 |
| Mutated | 47 (20.0) | 32 (21.9) | 15 (16.9) |  |
| ***NRAS*** |  |  |  |  |
| Wild-type | 73 (83.9) | 55 (87.3) | 18 (75.0) | 0.19 |
| Mutated | 14 (16.1) | 8 (12.7) | 6 (25.0) |  |
| **Place of birth** |  |  |  |  |
| North | 2 (0.9) | 1 (0.7) | 1 (1.3) | **<0.01** |
| Northeast | 75 (33.6) | 46 (31.9) | 29 (36.7) |  |
| Midwest | 30 (13.5) | 11 (7.6) | 19 (24.1) |  |
| Southeast | 95 (42.6) | 70 (48.6) | 25 (31.6) |  |
| South | 21 (9.4) | 16 (11.1) | 5 (6.3) |  |
| WBC: white blood cell; ALL: acute lymphoblastic leukaemia; AML: acute myeloid leukaemia; g: grams; n: number of cases. | | | | |
