# Supplementary material for: RAS mutations in early age leukaemia modulated by NQO1 rs1800566 (C609T) are associated with second-hand smoking exposures
Source: BMC Cancer. 2014 Feb 26;14:133. doi: 10.1186/1471-2407-14-133 (PMC3946262; doi:10.1186/1471-2407-14-133)
Supplement: Additional file 2: Table S2 — Demography and laboratorial characteristics of earl age leukaemia according to age groups, Brazil. [file 1471-2407-14-133-S2.doc]

|  |  | **Age (months)** | |  |
| --- | --- | --- | --- | --- |
|  | **Total, n(%)** | **≤12, n(%)** | **13-24, n(%)** | ***p*** |
| **Gender** |  |  |  |  |
| Male | 131 (55.7) | 69 (57.5) | 62 (53.9) | 0.58 |
| Female | 104 (44.3) | 51 (42.5) | 53 (46.1) |  |
| **Skin colour** |  |  |  |  |
| White | 139 (59.9) | 75 (63.0) | 64 (56.6) | 0.32 |
| Non-White | 93 (40.1) | 44 (37.0) | 49 (43.4) |  |
| **WBC (x10^9^/L)** |  |  |  |  |
| ≤50 | 106 (46.5) | 49 (43.0) | 57 (50.0) | 0.28 |
| >50 | 122 (53.5) | 65 (57.0) | 57 (50.0) |  |
| ***MLL* status** |  |  |  |  |
| Rearranged | 102 (48.3) | 62 (57.4) | 40 (38.8) | **<0.01** |
| Wild-type | 109 (51.7) | 46 (42.6) | 63 (61.2) |  |
| ***KRAS*** |  |  |  |  |
| Wild-type | 188 (80.0) | 95 (79.2) | 93 (80.9) | 0.74 |
| Mutated | 47 (20.0) | 25 (20.8) | 22 (19.1) |  |
| ***NRAS*** |  |  |  |  |
| Wild-type | 73 (83.9) | 38 (80.9) | 35 (87.5) | 0.40 |
| Mutated | 14 (16.1) | 9 (19.1) | 5 (12.5) |  |
| ***BRAF*** |  |  |  |  |
| Wild-type | 124 (99.2) | 59 (98.3) | 65 (100.0) | 0.48 |
| Mutated | 1 (0.8) | 1 (1.7) | 0 (0.0) |  |
| ***FLT3*** |  |  |  |  |
| Wild-type | 148 (93.1) | 80 (94.1) | 68 (91.9) | 0.58 |
| Mutated | 11 (6.9) | 5 (5.9) | 6 (8.1) |  |
| **NQO1 (rs1800566)^a^** |  |  |  |  |
| CC | 97 (54.2) | 45 (49.5) | 52 (59.1) | 0.24 |
| CT | 70 (39.1) | 41 (45.1) | 29 (33.0) |  |
| TT | 12 (6.7) | 5(5.5) | 7 (8.0) |  |
| ^a^ Genotype frequencies of *NQO1* polymorphism. WBC: white blood cell; ALL: acute lymphoblastic leukaemia; AML: acute myeloid leukaemia; n: number of cases. | | | | |

**Additional File 2: Table S2 Demography and laboratorial characteristics of earl age leukaemia according to age groups, Brazil**
