# Supplementary material for: RAS mutations in early age leukaemia modulated by NQO1 rs1800566 (C609T) are associated with second-hand smoking exposures
Source: BMC Cancer. 2014 Feb 26;14:133. doi: 10.1186/1471-2407-14-133 (PMC3946262; doi:10.1186/1471-2407-14-133)
Supplement: Additional file 3: Table S3 — The demography and laboratorial differences in the distribution of RAS mutations, according to age strata, Brazil, 2000-2010. [file 1471-2407-14-133-S3.doc]

|  | **≤12 months** | | | | |  | **13-24 months** | | | | |  |
| --- | --- | --- | --- | --- | --- | --- | --- | --- | --- | --- | --- | --- |
|  | ***KRAS*** | |  | ***NRAS*** | |  | ***KRAS*** | |  | ***NRAS*** | |  |
|  | **wt n(%)** | **mut n(%)** | ***p*** | **wt n(%)** | **mut n(%)** | ***p*** | **wt n(%)** | **mut n(%)** | ***p*** | **wt n(%)** | **mut n(%)** | ***p*** |
| **Gender** |  |  |  |  |  |  |  |  |  |  |  |  |
| Male | 56 (58.9) | 13 (52.0) | 0.53 | 26 (68.4) | 6 (66.7) | 1.00 | 53 (57.0) | 9 (40.9) | 0.17 | 24 (68.6) | 4 (80.0) | 1.00 |
| Female | 39 (41.1) | 12 (48.0) |  | 12 (31.6) | 3 (33.3) |  | 40 (43.0) | 13 (59.1) |  | 11 (31.4) | 1 (20.0) |  |
| **Skin colour** |  |  |  |  |  |  |  |  |  |  |  |  |
| White | 59 (62.8) | 16 (64.0) | 0.91 | 24 (63.2) | 7 (77.8) | 0.69 | 51 (56.0) | 13 (59.1) | 0.79 | 21 (61.8) | 4 (100.0) | 0.27 |
| Non-White | 35 (37.2) | 9 (36.0) |  | 14 (36.8) | 2 (22.2) |  | 40 (44.0) | 9 (40.9) |  | 13 (38.2) | 0 (0.0) |  |
| **WBC (x10^9^/l)** |  |  |  |  |  |  |  |  |  |  |  |  |
| ≤50 | 40 (44.9) | 9 (36.0) | 0.42 | 18 (52.9) | 3 (33.3) | 0.45 | 48 (52.2) | 9 (40.9) | 0.34 | 22 (62.9) | 1 (20.0) | 0.14 |
| >50 | 49 (55.1) | 16 (64.0) |  | 16 (47.1) | 6 (66.7) |  | 44 (47.8) | 13 (59.1) |  | 13 (37.1) | 4 (80.0) |  |
| **Leukaemia Subtypes** |  |  |  |  |  |  |  |  |  |  |  |  |
| ALL | 63 (66.3) | 19 (76.0) | 0.35 | 25 (65.8) | 8 (88.9) | 0.24 | 52 (55.9) | 16 (72.7) | 0.14 | 21 (60.0) | 3 (60.0) | 1.00 |
| AML | 32 (33.7) | 6 (24.0) |  | 13 (34.2) | 1 (11.1) |  | 41 (44.1) | 6 (27.3) |  | 14 (40.0) | 2 (40.0) |  |
| ***MLL* status** |  |  |  |  |  |  |  |  |  |  |  |  |
| Rearranged | 41 (48.8) | 21 (87.5) | **<0.001** | 19 (54.3) | 6 (66.7) | 0.71 | 34 (40.5) | 6 (31.6) | 0.47 | 9 (30.0) | 1 (20.0) | 1.00 |
| Wild-type | 43 (51.2) | 3 (12.5) |  | 16 (45.7) | 3 (33.3) |  | 50 (59.5) | 13 (68.4) |  | 21 (70.0) | 4 (80.0) |  |
| ***NQO1* (rs1800566)^a^** |  |  |  |  |  |  |  |  |  |  |  |  |
| CC | 33 (47.8) | 12 (54.5) | 0.58 | 18 (54.5) | 4 (44.4) | 0.71 | 40 (57.1) | 12 (66.7) | 0.46 | 18 (60.0) | 3 (60.0) | 1.00 |
| CT+TT | 36 (52.2) | 10 (45.5) |  | 15 (45.5) | 5 (55.6) |  | 30 (42.9) | 6 (33.1) |  | 12 (40.0) | 2 (40.0) |  |
| ^a^  Genotype frequencies of *NQO1* polymorphism. ALL: acute lymphoblastic leukaemia; AML: acute myeloid leukaemia; WBC: white blood cell; mut: mutated; wt: wild type; n: number of cases. | | | | | | | | | | | | |

**Additional File 3:Table S3 The demography and laboratorial differences in the distribution of *RAS* mutations, according to age strata, Brazil, 2000-2010.**
