# Supplementary material for: RAS mutations in early age leukaemia modulated by NQO1 rs1800566 (C609T) are associated with second-hand smoking exposures
Source: BMC Cancer. 2014 Feb 26;14:133. doi: 10.1186/1471-2407-14-133 (PMC3946262; doi:10.1186/1471-2407-14-133)
Supplement: Additional file 4: Table S4 — The association of RAS and FLT3 mutations in early age leukaemia according to MLL status, Brazil 2000-2010. [file 1471-2407-14-133-S4.doc]

**Additional File 4: Table S4. The association of *RAS* and *FLT3* mutations in early age leukaemia according to *MLL*** status, Brazil 2000-2010.

|  | **B-precursor ALL^a^** | | | | **AML** | | | | **Total Cases** | | | |
| --- | --- | --- | --- | --- | --- | --- | --- | --- | --- | --- | --- | --- |
|  | ***MLL^wt^***  **n (%)** | ***MLL^r^***  **n (%)** | ***p*** | **OR (95%CI)** | ***MLL^wt^***  **n (%)** | ***MLL^r^***  **n (%)** | ***p*** | **OR (95%CI)** | ***MLL^wt^***  **n (%)** | ***MLL^r^***  **n (%)** | ***p*** | **OR (95%CI)** |
| ***KRAS*** |  |  |  |  |  |  |  |  |  |  |  |  |
| Wild-type | 52 (81.2) | 50 (71.4) |  | 1^b^ | 39 (92.9) | 23 (79.3) |  | 1^b^ | 91 (85.8) | 73 (73.7) |  | 1^b^ |
| Mutated | 12 (18.8) | 20 (28.6) | 0.18 | 1.73 (0.77-3.91) | 3 (7.1) | 6 (20.7) | 0.14 | 3.39 (0.77-14.88) | 15 (14.2) | 26 (26.3) | **0.03** | 2.16 (1.07-4.38) |
| ***NRAS*** |  |  |  |  |  |  |  |  |  |  |  |  |
| Wild-type | 23 (82.1) | 19 (76.0) |  | 1^b^ | 14 (87.5) | 8 (88.9) |  | 1^b^ | 37 (84.1) | 27 (79.4) |  | 1^b^ |
| Mutated | 5 (17.9) | 6 (24.0) | 0.58 | 1.45 (0.38-5.51) | 2 (12.5) | 1 (11.1) | 1.00 | 1.00 (0.08-13.02) | 7 (15.9) | 7 (20.6) | 0.59 | 1.37 (0.43-4.37) |
| ***FLT3*** |  |  |  |  |  |  |  |  |  |  |  |  |
| Wild-type | 32 (88.9) | 50 (94.3) |  | 1^b^ | 29 (96.7) | 23 (92.0) |  | 1^b^ | 61 (92.4) | 73 (93.6) |  | 1^b^ |
| Mutated | 4 (11.1) | 3 (5.7) | 0.43 | 0.48 (0.10-2.28) | 1 (3.3) | 2 (8.0) | 0.58 | 2.52 (0.22-29.58) | 5 (7.6) | 5 (6.4) | 1.00 | 0.87 (0.23-3.02) |
| ^a^Seven T acute lymphoblastic leukaemia cases were excluded from this analysis. ^b^1 as a reference. ALL: acute lymphoblastic leukaemia; AML: acute myeloid leukaemia; *MLL^wt^*: Wild type *MLL*; *MLL****^r^***: Rearranged *MLL*; n: number of cases. | | | | | | | | | | | | |
