# Supplementary figures and images for: RAS mutations in early age leukaemia modulated by NQO1 rs1800566 (C609T) are associated with second-hand smoking exposures
Source: BMC Cancer. 2014 Feb 26;14:133. doi: 10.1186/1471-2407-14-133 (PMC3946262; doi:10.1186/1471-2407-14-133)

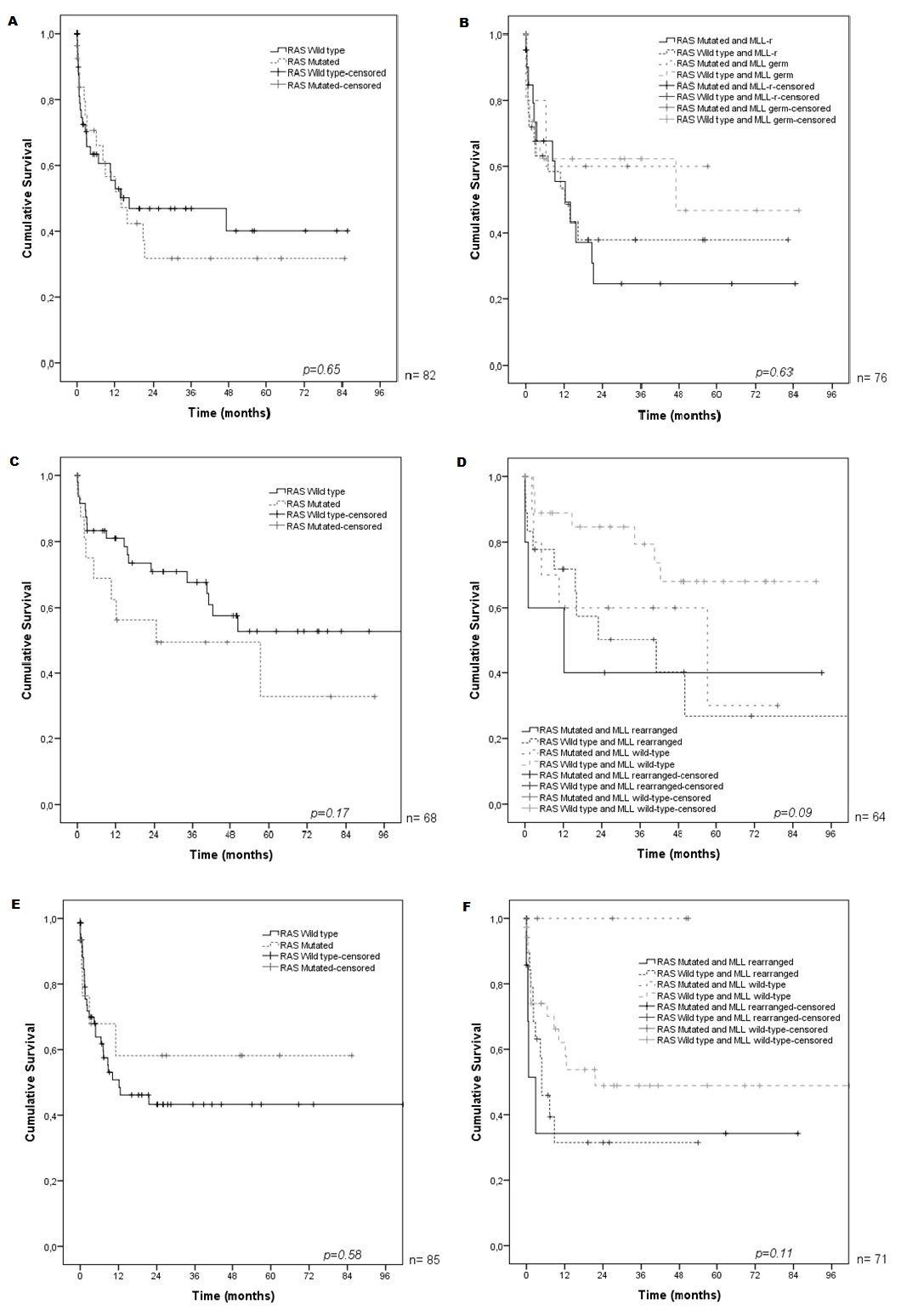

Supplement: Additional file 5: Figure S1 — Overall survival of RAS mutations (K- or N-RAS) and RAS (K- or N-RAS) wild-type among ALL and AML according to patients age at the diagnosis. (A) RAS mutations in ALL cases aged ≤12 months. (B) RAS mutations and MLL rearrangements in ALL cases aged ≤12 months. (C) RAS mutations in ALL cases aged 13–24 months. (D) RAS mutations and MLL rearrangements in ALL cases aged 13–24 months. (E) RAS mutations in AML cases aged ≤24 months. (F) RAS mutations and MLL rearrangements in AML cases aged ≤24 months. ALL: acute lymphoblastic leukaemia; AML: acute myeloid leukaemia. [file 1471-2407-14-133-S5.jpeg]
