# Supplementary material for: RAS mutations in early age leukaemia modulated by NQO1 rs1800566 (C609T) are associated with second-hand smoking exposures
Source: BMC Cancer. 2014 Feb 26;14:133. doi: 10.1186/1471-2407-14-133 (PMC3946262; doi:10.1186/1471-2407-14-133)
Supplement: Additional file 6: Table 5S — The frequency association between RAS mutations and NQ01 genotype in EAL. [file 1471-2407-14-133-S6.doc]

**Additional File 6 Table 5S The frequency association between *RAS* mutations and *NQ01* genotype in EAL**

|  | Total,  n (%) | ***RAS ^wt^* n(%)** | ***RAS ^mut^* n(%)** | **OR (95%CI)** | ***p*** |
| --- | --- | --- | --- | --- | --- |
| ***NQO1rs1800566*** |  |  |  |  |  |
| CC | 97 (54.2) | 67 (53.2) | 30 (56.6) | 1^a^ |  |
| CT | 70 (39.1) | 52 (41.3) | 18 (34.0) | 0.77 (0.39-1.54) | 0.46 |
| TT | 12 (6.7) | 7 (5.6) | 5 (9.4) | 1.60 (0.47-5.44) | 0.45 |
| CT+TT | 82 (45.8) | 59 (46.8) | 23 (43.4) | 0.87 (0.46-1.66) | 0.67 |
| ^a^ 1 as considered a reference; mut, mutated; wt, wild type. | | | | | |
