# Supplementary material for: RAS mutations in early age leukaemia modulated by NQO1 rs1800566 (C609T) are associated with second-hand smoking exposures
Source: BMC Cancer. 2014 Feb 26;14:133. doi: 10.1186/1471-2407-14-133 (PMC3946262; doi:10.1186/1471-2407-14-133)
Supplement: Additional file 7: Table S6 — The NQO1, RAS and MLL status in childhood leukaemia, Brazil 2000-2010. [file 1471-2407-14-133-S7.doc]

**Additional File 7: Table S6. The *NQO1*, *RAS* and *MLL* status in childhood leukaemia, Brazil 2000-2010.**

|  | ***NQO1^wt^* n(%)** | ***NQO1 609CT*** ^a^ **n(%)** | **OR (95%CI)** | ***p*** | ***RAS ^wt^* n(%)** | ***RAS ^mut^* n(%)** | **OR (95%CI)** | ***p*** |
| --- | --- | --- | --- | --- | --- | --- | --- | --- |
| **ALL** |  |  |  |  |  |  |  |  |
| *MLL^wt^* | 34 (51.5) | 20 (36.4) | 1^b^ |  | 50 (52.1) | 17 (38.6) | 1^b^ |  |
| *MLL^r^* | 32 (48.5) | 35 (63.5) | 1.86 (0.90-3.86) | 0.09 | 46 (47.9) | 27 (61.4) | 1.73 (0.83-3.57) | 0.14 |
| **AML** |  |  |  |  |  |  |  |  |
| MLL^wt^ | 16 (57.1) | 12 (60.0) | 1^b^ |  | 37 (62.7) | 5 (41.7) | 1^b^ |  |
| MLL^r^ | 12 (42.9) | 8 (40.0) | 0.89 (0.28-2.85) | 0.84 | 22 (37.3) | 7 (58.3) | 2.36 (0.67-8.33) | 0.21 |
| ^a^ *NQ01* genotype status included allele variants CT and TT. ^b^1 considered as reference. N: number of cases; wt: wild-type; mut: mutation; ALL: acute lymphoblastic leukaemia; AML: acute myeloid leukaemia; *MLL* gene; wt, wild-type; r, rearrangements. | | | | | | | | |
